# Direct Anterior Approach Using Navigation Improves Accuracy of Cup Position Compared to Conventional Posterior Approach

**DOI:** 10.7759/cureus.1482

**Published:** 2017-07-17

**Authors:** Jason Chow, Simon Pearce, Kuk-ki Cho, William Walter

**Affiliations:** 1 Orthopaedics, Nepean hospital; 2 Orthopaedics, Royal North Shore Hospital; 3 Orthopaedics, Mater Hospital

**Keywords:** orthopaedic surgery, total hip arthroplasty, total hip replacement, intraoperative navigation, arthroplasty, outcome measures

## Abstract

The accuracy of cup position in total hip arthroplasty is essential for a satisfactory result as malpositioning increases the risk of complications including dislocation, high wear rate, loosening, squeaking, edge loading, impingement and ultimately failure.

We studied 166 patients in a single-surgeon-series of matched cohorts of patients who underwent total hip arthroplasties. Four separate groups were identified comprising of the posterior approach +/- navigation and the direct anterior approach +/- navigation.

We found a significant difference between the direct anterior navigated group and the posterior non-navigated group for both anteversions (P < 0.05, confidence interval (CI) -3.86 to -1.73) and inclination (P < 0.05, CI -3.08 to -1.08). Almost, 72% of anterior navigated patients fell within 5^o^ of the navigation software set target cup position of 45^o^ inclination and 20^o^ anteversion and 100% were within 10^o^. Only 30% of posterior non-navigated were within 5^o^ of both anteversion and inclination and 73% were within 10^o^.

There was also a significant difference between the direct anterior navigated and non-navigated group with respect to anteversion only (p < 0.05, CI 1.50 to 1.30). There were no other significant differences between approaches +/- navigation.

The direct anterior approach allows ease of access to both anterior-superior iliac spines for navigation and a supine patient allows anteversion and inclination to be measured in the frontal plane. We conclude that the direct anterior approach with navigation improves the accuracy of cup position compared to the conventional posterior approach without navigation.

## Introduction

Improper positioning of the acetabular component in total hip arthroplasties (THA) has been shown to increase the dislocation rate [1-6], increase bearing surface wears [7-8], decrease the range of motion [9-10], increase revision rates [11] and contributes to squeaking [12]. Many factors have been described which contribute to accurate cup positioning. There are patient factors including body mass index (BMI) [13], age [14-16], gender [14, 16] and primary diagnosis for total hip arthroplasty (THA) [16-17]. There are surgical factors including the performing surgeon's experience [16, 18], the surgical approach [16, 19-24], the prosthetic components [14, 16, 25-28], the acetabular cup fixation method [17] and the orientation of the acetabular cup [14, 16, 27]. We designed this study to determine if there was a difference in acetabulum orientation when performed via the direct anterior approach (DAA) or the posterior approach (PA) with and without using navigation. We studied 166 patients comparing matched cohorts who underwent THA by the same surgeon at our institution. Four separate groups of patients were identified: Group 1 – PA with navigation; Group 2 – PA without navigation; Group 3 – DAA with navigation; Group 4 – DAA without navigation. Informed consent statement was obtained for this study.

## Materials and methods

A standardized statistical computer program developed by the University of Tennessee [29] was used to perform a prospective power calculation of a continuous response variable from cohorts containing the matched patients. Prior data from an unpublished pilot study indicated a sample size of 14 pairs of subjects to reject the null hypothesis that this response difference is zero with probability (power) 0.8. The Type I error probability associated with this test of this null hypothesis is 0.05.

All total hip arthroplasties performed by the senior author (WLW) from January 2004 to February 2013 were analyzed retrospectively. This included 650 PA THAs and 111 DAA. Only 13 of the PA group were navigated and only 21 of the DAA group were without navigation. Due to relatively small numbers in these two groups, the study was divided into two arms. Both arms matched patients according to gender, side, body mass index (BMI) and age. The first arm contained the 13 PA navigated group which was matched to 48 PA without navigation and 33 DAA with navigation (Table 1). The second arm contained the 21 DAA without navigation group which was matched to 26 PA without navigation and 25 DAA with navigation (Table 2).

**Table 1:** Table representing the demographics of the study's first arm

Category	NAV Posterior	NO NAV Posterior	NAV Anterior
Sex (Male:Female)	13 (8:5)	48 (24:24)	33 (10:23)
Body Mass Index	30	29	29
Age	65	70	71

**Table 2:** Table representing the demographics of the study's second arm

Category	NO NAV Anterior	NO NAV Posterior	NAV Anterior
Sex (Male:Female)	21 (4:17)	26 (5:21)	25 (7:18)
Body Mass Index	26	26	25
Age	66	68	68

All anteroposterior (AP) hip radiographs were exported or scanned into a validated computer program and the anteversion and inclination were measured.

## Results

**Figure 1:**
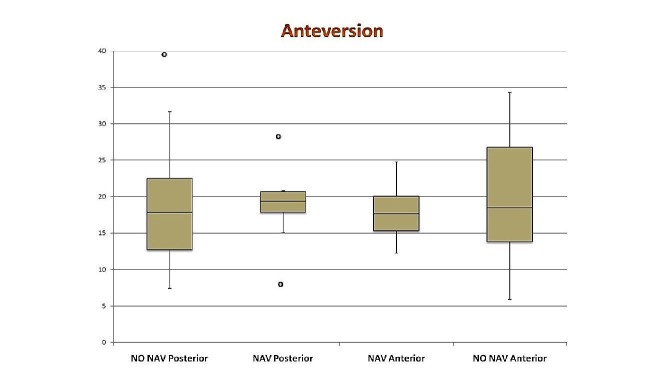
Box and whisker plot showing anteversion (degrees) in all four groups

**Figure 2:**
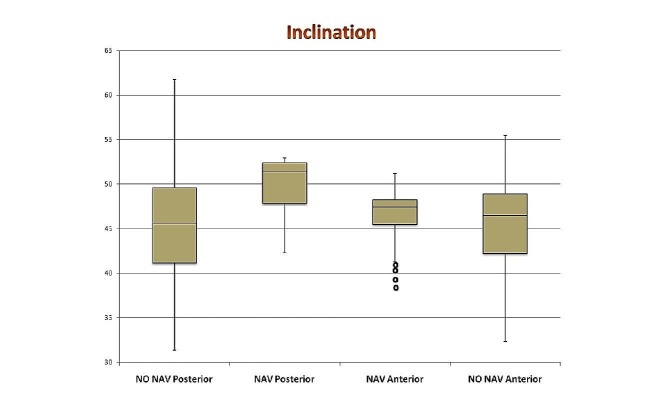
Box and whisker plot showing inclination (degrees) in all four groups

The results of mean anteversion and inclination for all four groups are shown in Table 3, in addition to the absolute difference of these values from a target of 20 degrees anteversion and 45 degrees inclination. Box and whisker plots for all four groups are shown in Figure 1 -2. A two-sample Student’s T-test for two independent means was used to compare cup position within each arm of the study. Consequently, no comparison can be made between PA navigated and DAA without navigation as they were in different arms of the study. We found a significant difference between the DAA navigated group and the PA non-navigated group for both anteversions (P < 0.05, CI -3.86 to -1.73) and inclination (P < 0.05, CI -3.08 to -1.08). Almost 72% of anterior navigated patients fell within 5 degrees of the target cup position of 45 degrees inclination and 20 degrees anteversion and 100% were within 10 degrees. Only 30% of posterior non-navigated were within 5 degrees of both anteversion and inclination and 73% were within 10 degrees.

**Table 3:** Table representing the cup position for all four groups and absolute difference from the target

Category	NAV Posterior	NO NAV Posterior	NAV Anterior	NO NAV Anterior
Anteversion	20^o^	18^o^	18^o^	20^o^
Absolute anteversion difference from 20^o^	4^o^	6^o^	3^o^	6^o^
Inclination	50^o^	46^o^	47^o^	46^o^
Absolute inclination difference from 45^o^	5^o^	5^o^	3^o^	5^o^

**Figure 3:**
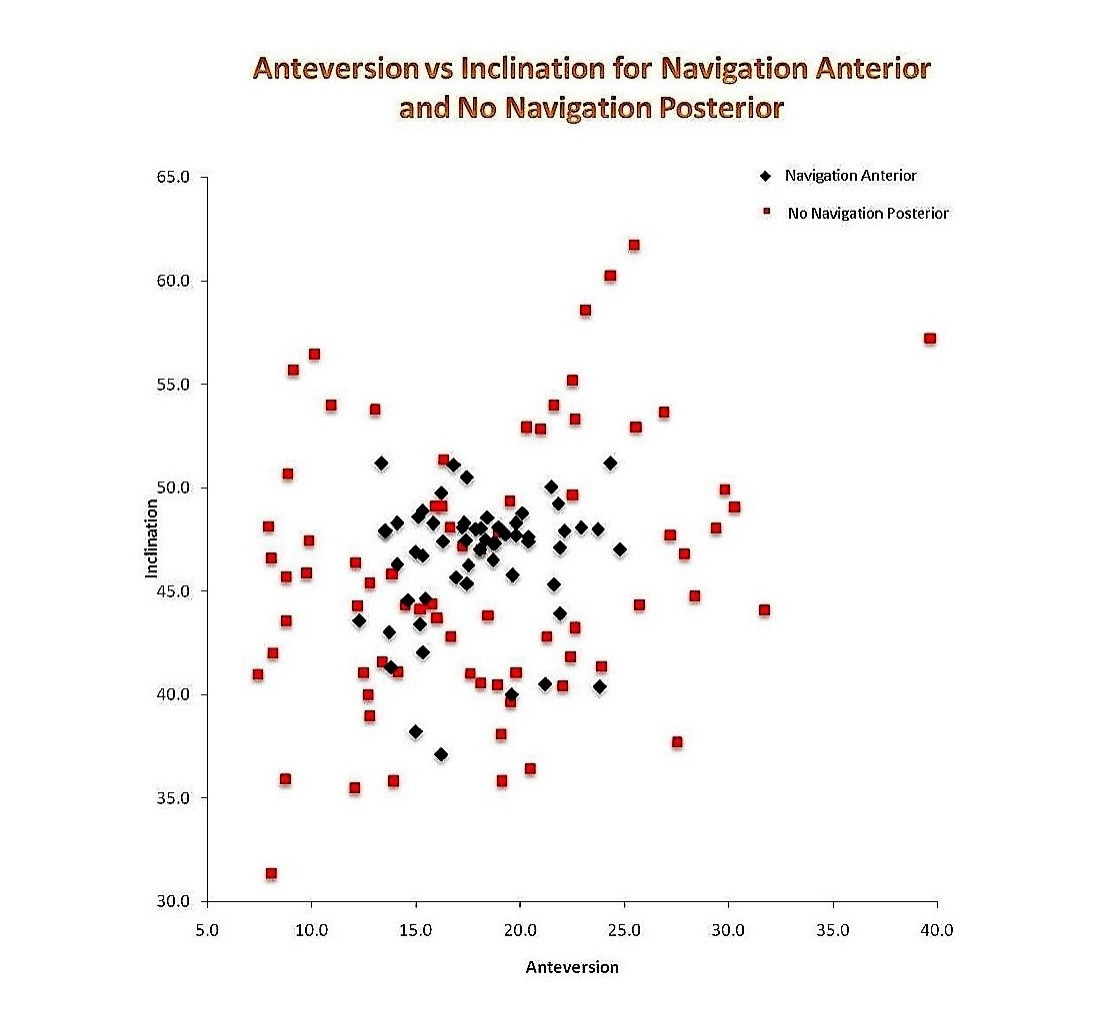
Scatterplot showing cup position of direct anterior approach (DAA) navigated vs posterior approach (PA) without navigation

There was also a significant difference between the direct anterior navigated and non-navigated group with respect to anteversion only (p < 0.05, CI 1.50 to 1.30). There were no other significant differences between approaches +/- navigation. The figure 3 shows a scatter plot comparing cup position between DAA navigated group and PA non-navigated group, which highlights the difference in precision. 

## Discussion

This study was designed to gain objective evidence of the accuracy of cup position whilst controlling as many of the variables as possible. All the patients were operated on by the same surgeon and all patients were rigorously matched with respect to gender, side, BMI, and age. The initial hypothesis was that the anterior approach allowed more accurate positioning of the cup for two reasons. Firstly, because it involves surgery in the frontal plane when the patient is supinely allowing an unobstructed view of both anterior superior iliac spines (ASIS) to assess the horizontal plane of the pelvis to reference off for inclination and the coronal plane of the pelvis can be inferred as being parallel to the floor to reference off for anteversion. Secondly, the supine position allows the contralateral ASIS to be prepared and draped allowing intraoperative navigation markers to be sited in a way not possible in a laterally positioned patient for the posterior approach.

There were several limitations to this study including the available numbers of patients in the two groups. The PA navigated and DAA non-navigated groups were small and did not allow for rigorous direct matching between the two groups. Since we could not draw statistical conclusion directly between these two groups, we elected to create two arms with rigorous matching between PA navigated and the two remaining groups (excluding DAA non-navigated) in one arm and the other arm containing DAA non-navigated and the two remaining groups (excluding PA navigated). This did not affect the comparison of PA non-navigated with DAA navigated as these were in the same arm of the study. Another potential limitation was the DAA non-navigated group included some cases in the first 30 DAA procedures that were performed by the senior author and therefore may be affected by the learning curve bias.

Our study showed that the DAA with navigation was significantly more accurate than the PA without navigation. The authors acknowledge that there is no robustly proven ideal position of the acetabular implant and the deviation from the target position of 45 degrees inclination and 20 degrees anteversion is based on the currently available literature and surgeon preference. Analysis of the level of deviation from this target revealed figures for outliers defined here as an absolute difference of greater than 10 degrees from the target position for both inclination and anteversion. Almost 72% of DAA navigated group was accurate within 5 degrees of the target and there were no outliers, compared to 30% of PA non-navigated group within 5 degrees and 27% were outliers.

## Conclusions

The direct anterior approach with navigation is more accurate when compared to the non-navigated posterior approach. It allows for improved visualization of the pelvic landmarks and along with the navigation, eliminates outliers and improves the accuracy of acetabular cup placement during total hip arthroplasties. The direct anterior approach with navigation allows for reproducible results and by decrease outliers hopefully, decreases the risk of potential complications related to the malposition of acetabular cups in total hip arthroplasties.
